# Effect of fixed orthodontic appliances on gingival status and oral microbiota: a pilot study

**DOI:** 10.1186/s12903-022-02511-9

**Published:** 2022-10-27

**Authors:** Zuzana Marincak Vrankova, Margarita Rousi, Michaela Cvanova, Daniela Gachova, Filip Ruzicka, Veronika Hola, Jan Lochman, Lydie Izakovicova Holla, Alena Brysova, Petra Borilova Linhartova

**Affiliations:** 1 Clinic of Stomatology, Institution Shared with St. Anne’s University Hospital, Faculty of Medicine, Masaryk University, Pekařská 53, 656 91 Brno, Czech Republic; 2 Clinic of Maxillofacial Surgery, Institution Shared with University Hospital Brno, Faculty of Medicine, Jihlavska 20, 625 00 Brno, Czech Republic; 3 RECETOX, Faculty of Science, Masaryk University, Kotlarska 2, Brno, Czech Republic; 4 Margarita Rousi Orthodontic Center, Athinon 91, 2035 Nicosia, Cyprus; 5 Institute of Biostatistics and Analyses, Faculty of Medicine, Masaryk University, Kamenice 5, 625 00 Brno, Czech Republic; 6 Department of Microbiology, Institution Shared with St. Anne’s University Hospital and Faculty of Medicine, Masaryk University, Pekařská 53, 656 91 Brno, Czech Republic; 7 Department of Biochemistry, Faculty of Science, Masaryk University, Kamenice 5, 625 00 Brno, Czech Republic

**Keywords:** Orthodontic treatment, Oral microbiota, Oral microbiome, *Candida* sp., Plaque index

## Abstract

**Background:**

This pilot study aimed to investigate how fixed orthodontic appliances simultaneously applied on the upper and lower arches affect the oral environment in the medium term.

**Methods:**

The oral status of 30 orthodontic patients was evaluated using the number of decay-missing-filled teeth (DMFT), plaque (PI), and gingival indices (GI) before bonding of fixed orthodontic appliances (T0) and during the therapy (T1). Besides, the gingival crevicular fluid (GCF) and a dental plaque were collected. Samples were analyzed for selected *Candida* sp. and for 10 selected oral bacteria using mass spectroscopy and multiplex polymerase chain reaction, respectively.

**Results:**

In 60% of patients, deterioration of the oral status (demonstrated by the increase in PI) was recorded (*p* < 0.05). Moreover, the changes in PI correlated with those of GI (*p* < 0.001). At the T1 time point, the mean representation of *Actinomyces* sp. in the total prokaryotic DNA in GCF and dental plaque of individual patients increased compared to T0 (*p* < 0.05). The probability of finding any of the 7 selected periodontal bacteria combined with *Candida* sp. was 10 times higher in patients in whom PI deteriorated between T0 and T1 (*p* < 0.01).

**Conclusions:**

Changes in the oral microbial diversity and an increase in PI were observed in the medium term after bonding of orthodontic appliance. Our study highlights the importance of a complex approach in this type of research as the association between clinical characteristics and combined microbial parameters is higher than when evaluated separately.

**Supplementary Information:**

The online version contains supplementary material available at 10.1186/s12903-022-02511-9.

## Background

Orthodontic treatment is one of the most common dental treatments in children; nevertheless, thanks to the innovative technology and new techniques, it is increasingly used in adults as well. It aims to ensure a functional and stable occlusion with an ideal aesthetic outcome [1]. However, as orthodontic treatment, especially fixed orthodontic appliances, takes usually at least 1 year (and often multiple years), it has the potential to affect, besides the teeth position, also oral health in general.

The placement of fixed orthodontic appliances compromises the patients’ oral environment through the presence of additional surfaces and impeding oral hygiene procedures, which can affect the oral microbiota balance [2]. Patients undergoing orthodontic treatment should be aware of this risk and follow the appropriate measures to prevent them.

Oral microorganisms are part of the natural environment of the oral cavity; they can, however, also become etiological agents of pathology [3, 4]. A shift in the composition of oral microbiota is, among other things, associated with diet (e.g., frequent carbohydrate intake) and poor oral hygiene; in effect, it can lead to an increased risk of developing dental caries or periodontal disease [5, 6].

Several studies found an increase in the representation of cariogenic bacteria (such as *Streptococcus mutans* and *Lactobacillus* sp.) and of potentially pathogenic gram-negative bacteria in patients undergoing orthodontic treatment [5, 7–12]. The gingival health could be also compromised during orthodontic therapy [13].

*Candida* is a commensal, harmless form of fungi that can be found in the oral cavity of 53% of the general population; however, if disturbances in the balance of microflora or debilitation of the host occur, it can also become invasive and pathogenic [14]. During orthodontic treatment, candida colonization as well as the representation of individual strains or species seems to vary over time [8, 9, 11, 15–20]. Recently, a review by Contaldo et al. emphasized that the prevalence of *Candida* sp., viruses, and protozoa in the oral microbiota of orthodontic patients remains unclear [7].

Quite a few studies have investigated oral dysbiosis in association with orthodontic appliances (see Table S1); complex analyses of microbiota including fungi, cariogenic as well as periodontal bacteria in the context of oral indices, which objectivize oral hygiene and oral status, are, however, exceedingly rare. To the best of our knowledge, only one such study has been published so far [19]. Its results are very valuable, as it describes the development of the microbiome until 90 days after the bonding of the orthodontic appliances. Considering that treatment with orthodontic appliances usually lasts many months to several years, the lack of complex information in the medium or long term represents a major gap in the current knowledge.

The presented study also aims to analyze how fixed orthodontic appliances simultaneously applied on the upper and lower arches in a controlled group acquired through relatively strict inclusion/exclusion criteria affect the oral environment in the medium term; in particular, it aims to determine the representation of oral candida and selected oral bacteria associated with oral dysbiosis, especially with dental caries and periodontitis, and to analyze changes in the oral hygiene and oral status of orthodontic patients, measuring DMFT (decay-missing-filled teeth) index, plaque (PI) and gingival indices (GI). In addition, it also aims to evaluate the potential associations between the oral microbiota and dental hygiene status during orthodontic treatment.

## Methods

### Subjects, inclusion, and exclusion criteria

Patients who were offered participation in this prospective cohort study were selected from the pool of patients treated at the Orthodontic Department at the Clinic of Stomatology of the St. Anne’s University Hospital Brno, Czech Republic. The patients and/or their legal custodians were informed about all aspects of the research study and voluntarily confirmed their willingness to participate. A questionnaire and informed consent were given to all patients and/or their parents/legal custodians. Informed consent was signed before any examination was performed.

The inclusion criteria for participation in the study were: i) Czech nationality; ii) Being referred to the Orthodontic Department of the St. Anne’s University Hospital in Brno for the treatment of both the upper and lower jaw with fixed orthodontic appliance.

The general exclusion criteria for participation in the study were: i) age less than 10 and more than 30 years; ii) history of systemic diseases (diabetes mellitus, obesity, cardiovascular diseases, oncological diseases, immunodeficiency) or any other condition that can influence the oral microbiota or periodontal supporting tissues, such as alcoholism; iii) pregnancy or breastfeeding; iv) history of antibiotic administration in the last 2 months before the 1st examination and/or at any time during the study.

Additional orthodontic exclusion criteria were: i) initial poor oral hygiene contraindicating the bonding of fixed appliances (indicated by gingivitis or significant presence of tartar); ii) previous orthodontic treatment; iii) patient’s unwillingness to come for the second examination by the end of the 7^th^ month after the first examination; iv) patients who didn’t have both upper and lower fixed appliance bonded during one appointment; v) change of the attending orthodontist during the treatment.

### Questionnaires and clinical examination

Patients filled in the questionnaire including questions about smoking, alcohol, diet, taste preferences, and oral hygiene. These data were collected as a part of a different study.

The smoking status was recorded; individuals who never smoked were referred to as non-smokers, and both former and current smokers were referred to as smokers. The overall status of oral hygiene (classified as excellent, good, or poor oral hygiene) was evaluated also clinically.

The clinical evaluation and sample collection were done at two time points; before the bonding of fixed orthodontic appliances (T0) and during the treatment, by or before the end of the 7^th^ month of the treatment (T1).

All patients underwent a complex initial intra- and extraoral examination including a panoramic radiograph. After the examination, the diagnosis was established, and the therapeutic approach set.

The DMFT index was calculated in both T0 and T1 time points, assessing the dental caries prevalence of each patient. Besides, gingival and plaque status were examined at T0 and T1 time points, and PI and GI were calculated.

PI is based on recording both soft debris and mineralized deposits on teeth 12, 16, 24, 32, 36, and 44. Each of the four surfaces of the teeth (buccal, lingual, mesial, and distal) is given a score from 0 to 3 (0 = no plaque; 1 = a film of plaque adhering to the free gingival margin and adjacent area of the tooth; 2 = moderate accumulation of soft deposits within the gingival pocket of the tooth; 3 = abundance of soft matter within the gingival pocket and/or on the tooth). PI of a tooth is calculated as the mean score from its four sides [21].

The GI was created for the assessment of the gingival condition and reflects qualitative changes in the gingiva. It separately scores the marginal and interproximal tissues using a WHO (World Health Organisation) periodontal probe (0 = normal gingiva; 1 = mild inflammation; 2 = moderate inflammation; 3 = severe inflammation). GI for any individual tooth is then calculated as the mean score from the four areas (buccal, lingual, mesial, and distal) of the tooth [21].

### Orthodontic treatment

OmniArch^®^ brackets (Dentsply Sirona Orthodontics Inc., Sarasota FL 34243, US) were used for orthodontic treatment. After bonding using Transbond XT (3M, Unitek TM, Monrovia, CA, USA), BioStarter 0.012 in. or 0.014 in. (Forestadent, Pforzheim, Germany) were used as the first archwire for alignment. All patients (and patient’s parents in the case of underage children) were instructed on the proper dental hygiene during the orthodontic therapy at the beginning of the treatment and this was affirmed during subsequent follow-ups were sufficiently educated about oral hygiene before the bonding of fixed orthodontic appliances.

### Samples collection

A dental plaque sample was collected from the first upper right molar (16) using a sterile swab (COPAN Italia, Microbiology swab Transystem™). The collected swab was immediately placed in Amies medium (COPAN Italia, SpA, Brescia, Italy) and transported for analysis of *Candida* sp.

Another sample of the gingival crevicular fluid (GCF) and dental plaque was collected from the lower right first molar (46) using a paper cone (ISO 40) for detection of selected cariogenic and periodontal bacteria. The paper cone was immediately placed into a 1.5 mL sterile microtube and stored at a temperature of − 70 °C.

### Analysis of oral microbiota

The presence of *Candida* sp. in the samples of the dental plaque was analyzed in the laboratory of the Department of Microbiology, St. Anne’s University Hospital in Brno, Czech Republic. The samples were inoculated on Sabouraud 4% maltose agar (Merck KGaA, Darmstadt, Germany) and incubated for 48 hours at 37 °C. Identification of isolated strains was performed using the MALDI TOF-MS (Matrix-assisted laser desorption ionization-time of flight mass spectrometry). Samples were processed by the extended direct transfer method according to the manufacturer’s instructions (Bruker Daltonics, Billerica, MA, USA; MALDI Biotyper Protocol Guide; Edition 2, 2014). Individual colonies were applied as a thin film onto a target of the MALDI 96-target plate (Bruker Daltonics, Billerica, MA, USA and were overlaid with 1 μL formic acid. The dried sample was overlaid with 1 μL of the matrix solution (saturated α-cyano-4-hydroxycinnamic acid; Bruker Daltonics, Billerica, MA, USA) in the acetonitrile-water-trifluoroacetic acid (50:47.5:2.5, v/v) mixture, and allowed to dry before testing.

MALDI-TOF MS measurements were carried out with a MALDI BioTyper system (Bruker Daltonics, Billerica, MA, USA) and FlexControl 3.4 software (Bruker Daltonics, Billerica, MA, USA). Mass spectra were processed using BioTyper 3.1 software (Bruker Daltonics, Billerica, MA, USA).

The manufacturer-recommended cut-off scores were used for identification, with scores of ≥2.000 indicating identification to the species level, scores between 1.700 and 1.999 indicating identification to the genus level, and scores of < 1.700 indicating no identification. Isolates producing scores of < 1.700 were retested, and the higher of the scores were used for identification.

Microbial analysis of 3 cariogenic bacteria (*S. mutans*, *Lactobacillus* sp., *Actinomyces* sp.) and 7 periodontal bacteria (*Aggregatibacter actinomycetemcomitants*, *Tannarella forsythia*, *Porphyromonas gingivalis*, *Prevotella intermedia*, *Treponema denticola*, *Parvimonas micra*, *Fusobacterium nucleatum*) was done by multiplex real-time PCR (qPCR) as described previously [22]. Levels of cariogenic and periodontal bacteria were evaluated using the percentage of the total bacteria in the GCF and dental plaque of the patient.

### Statistical analysis

All statistical analyses were performed in IBM SPSS Statistics for Windows, version 26, unless otherwise stated. The significance level was set at 0.05.

As BMI values valid for adults cannot apply to adolescents, the BMI of adolescents was transposed to a z-score, which was in turn converted to a percentile. Z-score of BMI for age and sex was calculated in the Epi Info TM 7.2.4.0 software (Centers for Disease Control and Prevention (CDC) in Atlanta, Georgia, USA) according to WHO reference 2007 (5–19 years) [23]. Where needed, the difference between the measurements at the T0 and T1 time points was calculated by simple T1 − T0 subtraction. To simplify the calculations and interpretation, selected parameters were dichotomized. If a particular parameter improved or didn’t change in T1 in comparison with T0 in the patient, the value “unchanged or improved” was assigned. If the specific parameter value decreased in T1 in comparison with T0, the change was classified as “deterioration”.

The differences in plaque and gingival indexes (PI, GI) between T1 and T0 measurements, as well as differences in the prokaryome, were evaluated by Wilcoxon matched-pairs signed-rank test. For evaluation of the overall differences between the binarized groups, logistic regression with odds ratios (OR) was used. For the comparison of a number of the patients with deterioration of PI and GI in groups of prokaryome status changes, Fisher’s exact test was applied. Spearman’s correlation coefficient was used to express relationships between the age, DMFT, PI, and GI.

## Results

### Demographic data

**Fig. 1:**
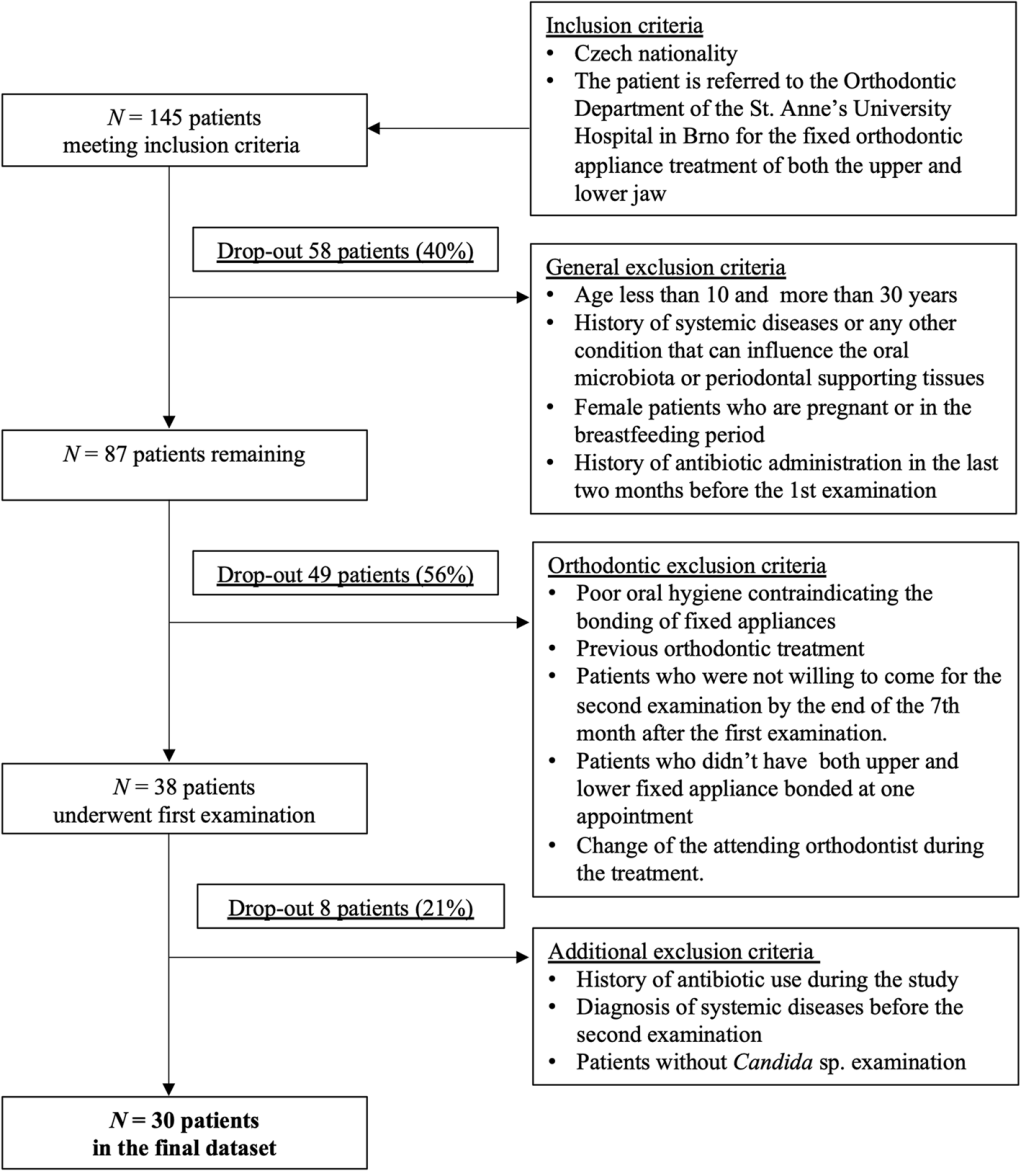
Flowchart of inclusion and exclusion criteria for the pilot study

Out of a total of 145 patients originally participating in the study, only 30 patients met all inclusion and exclusion criteria and were included in the evaluation (see the flowchart in Fig. 1). 47% of this final cohort were males and the mean (median) age of the patients was 16.8 (14.8) years at the T0 time point.

According to the results from the questionnaire distributed among the patients, BMI (body mass index) values were with a mean (median) BMI percentile of 53.2 (48.7) in adolescents (*N* = 21) and BMI of 25.1 (25.3) in adults (*N* = 9). BMI in adolescents was converted to percentiles to facilitate the correction for age and sex in juvenile patients.

87% of the patients were non-smokers, 2 patients were former smokers, and 2 patients were current smokers smoking more than 5 cigarettes per day. 45% of patients stated that sweet was their favourite taste and 43% usually sweetened their drinks. 30% of patients drank occasionally alcohol and the same percentage of patients drank also energetic drinks. However, except for the changes in the presence of *Candida* sp. in patients preferring sweet taste, none of the other behavioral characteristics were significantly associated with PI, GI or changes in oral microflora (see Table S3).

Dental hygiene was good almost in all patients. According to the questionnaire, 87% of patients brushed their teeth at least twice a day and 60% attended regular dental preventive examinations twice a year. Nevertheless, 70% of these patients did not attend dental hygienists; on the other hand, 6% of patients underwent professional oral hygiene at least twice a year.

### Oral indices before and during orthodontic treatment

The mean (median) DMFT was 2 (1) and did not change between T0 and T1 (*p* > 0.05). The DMFT value correlated with age (r_S_ = 0.545, *p* = 0.002).

**Fig. 2:**
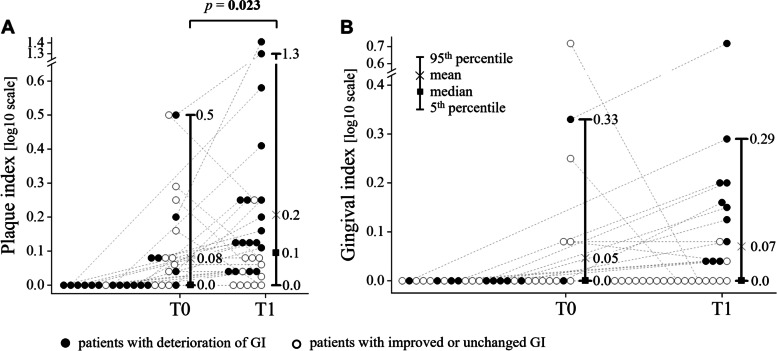
Differences in the plaque (**A**) and gingival (**B**) indices between two time-points – T0 (before bonding of fixed orthodontic appliances) and T1 (by or before the end of the 7^th^ month after bonding of fixed orthodontic appliances) in 30 patients. Full circle, patient with deterioration of clinical index; empty circle, patient with unchanged or improved clinical index; *p*-value: statistical significance was calculated using the Wilcoxon matched-pairs signed-rank test

Neither PI nor GI correlated with age at the initial examination (*p* > 0.05). During the first examination (i.e., before bonding of fixed orthodontic appliances, T0 time point), the mean values of PI and GI were 0.08 and 0.05, respectively. During the second examination (median 3.2 months, 2.7–6.5 months after bonding of fixed orthodontic appliances, T1), the mean values of PI and GI were 0.21 and 0.07, respectively. A significant difference between T1 and T0 time points was found in PI (*p* = 0.023) but none in GI (*p* = 0.116) values, see Fig. 2. The PI parameter deteriorated in 60% and GI in 37% of patients, respectively. A correlation between changes in GI and PI was observed (r_S_ = 0.614, *p* < 0.001).

### Oral microbiota before and during treatment with orthodontic appliances

**Fig. 3:**
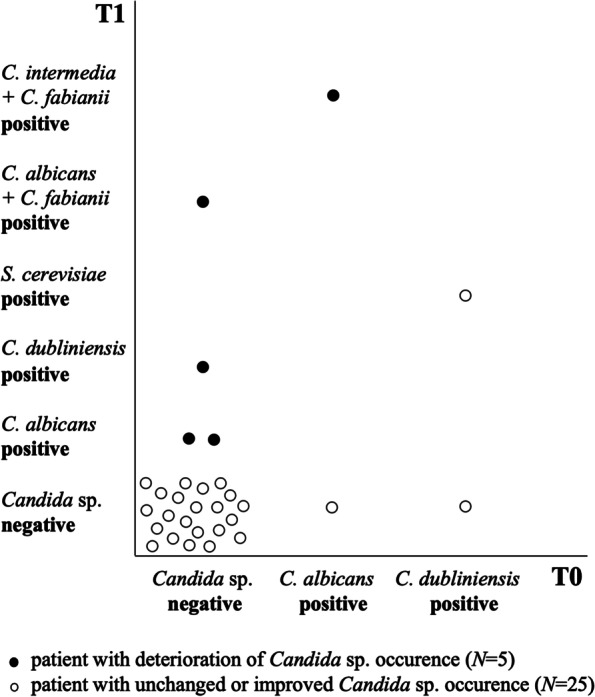
Occurrence of *Candida* sp. at two time points – T0 (before bonding of fixed orthodontic appliances) and T1 (7^th^ month after bonding of fixed orthodontic appliances) in 30 patients *C*., *Candida*; *S*., *Saccharomyces*

During the first analysis (T0), most of the patients (87%) were negative for *Candida* sp.; 7% of patients were positive for *Candida albicans* and 7% for *Candida dubliniensis*. During the follow-up (T1), most patients (80%) remained negative for *Candida* sp., but in half of the remaining 20% of patients other Candida species than *C. albicans* and *C. dubliniensis* were also detected, including *Cyberlindnera fabianii* (previously *Candida fabianii*)*, Candida intermedia,* and/or *Saccharomyces cerevisiae*, see Fig. 3*.* No significant difference in the presence of *Candida* sp. between T1 and T0 was detected (*p* > 0.05).

The mean (median) PI difference in patients negative for *Candida* sp. in both examinations was 0.05 (0.04) and GI mean (median) difference was 0.02 (0.00). The highest PIs and GIs were detected in patients in whom multiple *Candida* species were found in time T1; PI of 1.21 and GI of 0.33 were found in a patient with *C. fabianii* and *C. intermedia;* PI of 0.58 and GI of 0.28 were detected in another patient with *C. albicans* and *C. fabianii.*

**Fig. 4:**
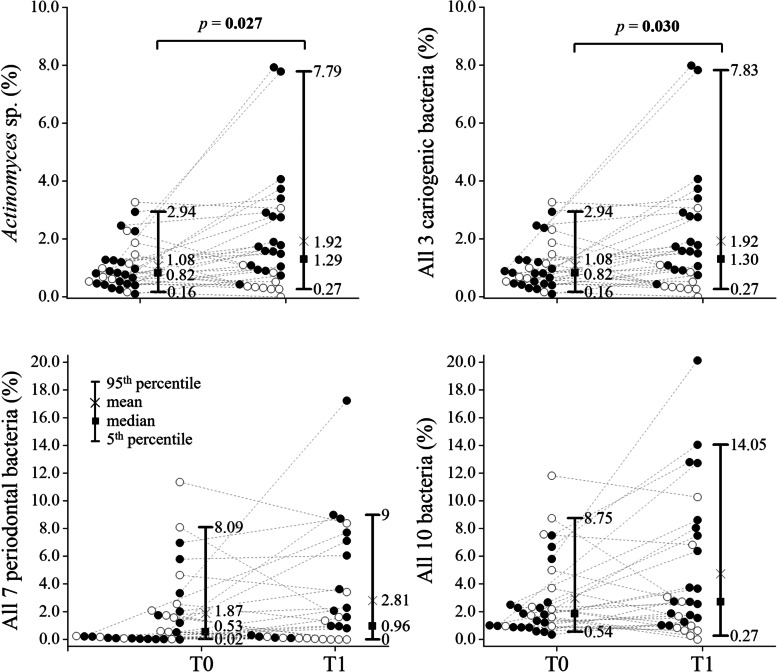
Differences in the percentages of 3 selected cariogenic (*Streptococcus mutans*, *Lactobacillus* sp., *Actinomyces* sp.) and 7 periodontal bacteria (*Aggregatibacter actinomycetemcomitants*, *Tannarella forsythia*, *Porphyromonas gingivalis*, *Prevotella intermedia*, *Treponema, Denticola*, *Parvimonas micra*, *Fusobacterium nucleatum*) relative to the total prokaryotic DNA at two time points – T0 (before bonding of fixed orthodontic appliances) and T1 (till the end of 7^th^ month after bonding of fixed orthodontic appliances) in 30 patients. Full circle, patient with deterioration of oral bacterial composition; empty circle, patient with unchanged or improved oral bacterial composition; statistical significance was calculated using the Wilcoxon matched-pairs signed-rank test

The findings of cariogenic bacteria *S. mutans* and *Lactobacillus* sp. in GCF/dental plaque samples at T0 and T1 time points were similar (*p* > 0.05). However, a significant difference between time points was detected in the percentage of *Actinomyces* sp. (i.e., the share of *Actinomyces* sp. in the total bacterial DNA) in the GCF/dental plaque (*p* = 0.027), see Fig. 4 and Table S3. Thus, *Actinomyces* sp. were the driver of the difference observed for the sum of three cariogenic bacteria in GCF/dental plaque between T1 and T0 (*p* = 0.030).

The percentage of neither any of the 7 periodontal bacteria nor their sum in the GCF/dental plaque samples differed significantly between T1 and T0 (*p* > 0.05). Similarly, the percentage of the sum of all 10 studied bacteria in the total prokaryotic DNA did not significantly change (borderline insignificant *p* = 0.086) between time points, see Fig. 4 and Table S3.

As far as the presence or absence of bacteria in individual samples is concerned, *F. nucleatum* was the most common (present in 97% of patients at T0 and 90% of patients at T1).

In the binary evaluation (the higher percentage vs. same or lower percentage of this bacteria in time points T1-T0), the association of the differences in the relative abundance of *F. nucleatum* and PI (deterioration vs. unchanged or improved state) was borderline insignificant (*p* = 0.061). The percentage of bacteriome represented by *F. nucleatum* increased in 72% of patients whose PI got worse, but only in 33% of patients whose PI didn’t. The same result was noted for the combined total of 7 periodontal bacteria (*p* = 0.061, Fisher’s exact test).

**Fig. 5:**
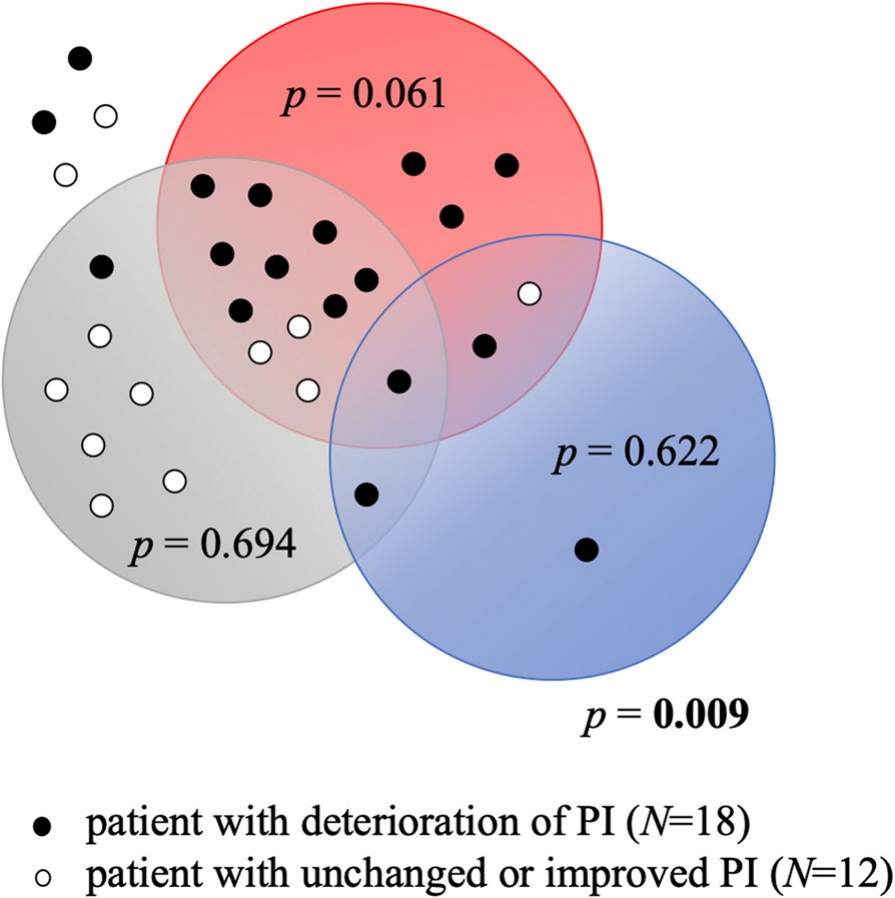
Changes in plaque indices of 30 orthodontically treated patients and in their oral microbial status; i.e., the association of changes in plaque index (PI) with the deterioration of the *Candida* sp. status (i.e., increase in the number of *Candida* sp.), of the percentage of 7 periodontal bacteria, and of the percentage of 3 selected cariogenic bacteria relative to the total prokaryotic DNA between T0 and T1 time points; blue circle, patients with deterioration of *Candida* status; red circle, patients with deterioration of the status of the 7 periodontal bacteria (*Aggregatibacter actinomycetemcomitants*, *Tannarella forsythia*, *Porphyromonas gingivalis*, *Prevotella intermedia*, *Treponema, Denticola*, *Parvimonas micra*, *Fusobacterium nucleatum*); grey circle, patients with deterioration of the status of the 3 cariogenic bacteria (*Streptococcus mutans*, *Lactobacillus* sp., *Actinomyces* sp.); *N*, number of patients; the differences between groups are evaluated using the Fisher’s exact test

In 18 patients, deterioration of PI was observed between T1 and T0, while in 12 PI remained unchanged or improved. The PI status (deterioration vs non-change or improvement) did not have any significant effect on the change in status of *Candida* sp. between T0 and T1 (*p* = 0.335); the same can be said about PI status and the sum of 3 cariogenic bacteria (*p* = 0.423)**.** There was a borderline insignificant difference in the percentage of the sum of 7 selected periodontal bacteria in the total prokaryotic genome in the GCF/dental plaque between these two groups (*p* = 0.061). This change is driven predominantly by the association between the PI change and the status of *F. nucleatum* alone, the most abundant bacteria in our sample. Moreover, a combined analysis (the deterioration of the sum of 7 selected periodontal bacteria together with *Candida* sp. vs. PI change) revealed that patients whose PI got worse had a 10 times higher probability of the deterioration in this microbial parameter than those in whom PI remained unchanged or improved (*p* = 0.009, OR 10.00, 95% CI 1.78–56.15), see Fig. 5.

## Discussion

Fixed orthodontic appliances increase the plaque-retentive space, leading to plaque buildup. As dental plaque is an important etiological factor for gingivitis, poor oral hygiene can increase the risk of gum inflammation [24]. Even though each orthodontic patient should be instructed on proper oral hygiene right at the beginning of the treatment, maintaining sufficient oral hygiene can prove challenging for these patients.

In this pilot study, the level of hygiene was evaluated during the follow-ups to ensure the maximum homogeneity in the studied group. If there was a deterioration in the level of oral hygiene, patient was referred to a dental hygienist for further training and instruction. At each follow-up, a deep cleaning of teeth was performed after the examination and sample collection by ortodontist.

Despite this protocol, we observed a significant worsening of the PI score over the medium term after the bonding of orthodontic appliances. Although GI got also worse to a certain degree, this worsening was not statistically significant; however, the changes in GI correlate with PI changes. From these findings, we can conclude that the bonding of fixed orthodontic appliances negatively affected oral hygiene but clinical changes in gingival health were not observed. However, this observation can be limited by the short period of investigation. Boyd et al. reported changes in plaque accumulation during orthodontic treatment (observations over 10 months in 6-week intervals) and worsening of the indices [25]. In contrast to these observations, Bergamo et al. found no significant difference in PI and GI between the pre-application stage and short-term after the application of fixed orthodontic appliances (30, 60, and 90 days after bonding) [19].

In our study, the highest PIs and GIs during the treatment were observed in patients with multiple *Candida* species. According to our findings, the deterioration in the status of the sum of 3 cariogenic bacteria and *Candida* sp. in GCF/dental plaque samples was similar in the groups with and without PI deterioration but a significant difference was observed in the percentage of the sum of 7 selected periodontal bacteria. Also, combined aggravation of the 7 selected periodontal bacteria and *Candida* sp. was 10 times more likely in patients with PI deterioration. This could be a transitory effect that depends on oral hygiene. It is extremely important to keep orthodontic patients under strict control of oral hygiene and plaque accumulation to favour rebalancing between the host and microorganisms after the appliance placement. Proper oral hygiene minimizes the increase in plaque accumulation in orthodontic patients and, thus, the possibility of tooth decalcification and the development of inflammatory periodontal disease.

In 2017, Guo et al. published a systematic review and meta-analysis including 13 studies investigating microbial changes in subgingival plaque of orthodontic patients [26]. The authors concluded that during orthodontic treatment, the level of subgingival pathogens increases but that increase is often only temporary [26]. Several other studies investigated bacteria and/or *Candida* sp. in patients, see Table S1 [8–11, 13, 15–20, 27, 28]. The number of patients in these studies varied from 15 to 124 subjects aged 4 to 27 years. Four studies were short-term, observing the microbial changes for 1–3 months only; four studies analyzed the changes for 4–6 months and three more were long-term studies. Two studies were not specific about the timeline. Most of the studies used saliva samples and/or oral swabs for the identification of bacteria and *Candida* sp. The microbiota was identified by various methods including conventional microbiological methods, PCR, MALDI-TOF-MS, or checkerboard DNA-DNA hybridization.

The main limitation of these studies is that not all of them monitor clinical indices, such as GI and PI. A design similar to our study was employed by Bergamo et al., who evaluated clinical indices, GI and PI, as well as bacteria and *Candida* sp. in saliva samples at even more time points than we did, with the longest follow-up of 90 days and found a significant decrease in the total amount of *Candida* sp., the purple, red, green, yellow and orange complexes during the treatment. The *Porphyromonas gingivalis* was an exception and showed the highest levels of incidence at T3 [19]. Compared to that study however, our cohort (even the final one) is larger (30 vs 15 patients, respectively, in the final evaluation) and the age range in our study is also narrower than in ours (10–30 vs 11–40 years). Moreover, the microbial composition in our study is analyzed from plaque samples while in their study, non-stimulated saliva was sampled [19]; microbiome in dental plaque and GCF samples reflect the oral hygiene in patient more and are directly related to clinical oral indices (PI and GI).

In our study, we used a highly selective and sensitive multiplex qPCR for identification of selected oral bacteria. This method has a possible downside for certain applications (i.e., the capability to detect also nucleic acids from non-viable microorganisms). This, however, does not apply to the presented study as we did not analyze any effects of antimicrobials but rather investigated bacterial composition in relation to clinical indices and as bacterial DNA is relatively quickly degraded in this environment, even detection of non-viable bacterial DNA still reflects the current and/or very recent composition of oral bacteria.

During our first pre-treatment analysis, most of the patients were negative for *Candida* sp. During orthodontic treatment, 10% of patients were positive besides *C. albicans* and *C. dubliniensis* also for other species including *C. fabianii, C. intermedia,* or *S. cerevisiae.* Our study supports the findings by Tapia et al. and Sanz-Orio-Soller et al., who detected no statically significant changes in *Candida* sp. colonization during the orthodontic treatment [16, 17].

Klaus et al.detected *Candida* sp. in all patients within his study; however, the prevalence was higher in patients with poor oral hygiene and white spot lesions [9]. Hernández-Solíse et al. reported an increased presence of *Candida* sp. 6 months after bonding of orthodontic appliances compared to the pre-bonding period; in 20% of patients in their study, *C. tropicalis* presence was detected [15]. Also, Perkowski et al. and Grzegocka et al. concluded that orthodontic treatment promotes *Candida* sp. colonization, which correlates with the application of fixed appliances and varies over time [8, 11]. Zheng et al. reported a significant increase in both the percentage of patients with candidiasis and in the number of colony-forming units (CFU) of *Candida* sp. 2 months after the application of fixed orthodontic appliances, followed by a decrease over time [18]. Interestingly, an increase in *Candida* sp. was also found in patients treated with removable orthodontic appliances [29]. Bergamo et al. reported a general decrease in the levels of *Candida* sp. 60 days after the brackets bonding; however, a decrease in *C. albicans* representation was observed only after 90 days [19].

In 2018, Lucchesse et al. published a systematic review concluding that orthodontic appliances of any type lead to an increase in the counts of *S. mutans, Lactobacillus* sp., and potentially pathogenic gram-negative bacteria, with a significant change observed as soon as after 1 month of treatment [12]. Arab et al. examined 30 orthodontic patients before the therapy and after 6, 12, and 18 weeks of treatment. Their study showed that the number of colonies of *S. mutans* and *Lactobacillus acidophilus* increased at 6 and 12 weeks after the bonding of fixed orthodontic appliances but after that, a decrease was observed [28]. Klaus et al. described the presence of *S. mutans* and *Lactobacilli* in all orthodontically treated patients included in their study with the duration of therapy ranging between 13.4–19.6 months. Patients with poor oral hygiene and white spot lesions showed an even higher prevalence of *Lactobacilli* [9]. Perkowski et al. detected various potentially pathogenic bacteria from the *Streptococcus* and *Enterococcus* groups. This is in agreement also with other studies in which the authors found an increase in the counts of *S. mutans* and *Lactobacillus* sp. or in the percentage of potentially pathogenic gram-negative bacteria during the orthodontic treatment [5, 8, 10, 20].

In our study, the percentage of 3 cariogenic bacteria in GCF and dental plaque differed between T1 and T0 time points. The percentage representation of *S. mutans* and *Lactobacillus* sp. in the oral microbiota of the GCF/dental plaque samples was similar before and during the treatment; however, the percentage of *Actinomyces* sp. increased during the treatment.

Kouvelis et al. found an increase in the number of CFU of *S. oralis* after 30 days of therapy; after another 60 days, however, it decreased to the initial level. The number of CFU of *S. sanguinis* decreased in both measurements and that of *S. salivarius* increased after 90 days of therapy. In summary, the authors concluded that orthodontic treatment, especially its initial phase, may not be associated with significant changes in the oral microbiota [27].

Several studies analyzing the effects of bonding of orthodontic appliances from quantitative and/or qualitative perspectives described changes in the representation of periodontal bacteria, such as *A. actinomycetemcomitans*, *P. gingivalis*, *P. intermedia*, and *T. forsythia* [30–36]. However, studies investigating the effects over the long term or before the application and after the removal of orthodontic appliances generally agreed on the decrease or even complete return of periodontal pathogens to the pretreatment level [33, 35, 37–41]. Our study showed no significant changes in the quantity of selected oral periodontal microorganisms at T1 compared to T0. No significant changes in the representation of either of the 7 studied periodontal bacteria in GCF/dental plaque samples changed during the orthodontic treatment. The representation of *F. nucleatum* increased in 72% of patients whose PI got worse but only in 33% of those with unchanged/improved PI, which may indicate the association of the level of oral hygiene, represented by the PI, with the abundance of *F.nucleatum*.

In 1997, Paolantonio et al. aimed to assess the differences in the periodontal status and in the occurrence of *A. actinomycetemcomitans* between patients wearing the orthodontic appliances and the control group. They concluded that the presence of orthodontic appliances led to an increase in PI indices as well as in positive isolation of *A. actinomycetemcomitans,* which they confirmed also in their follow-up study [32].

Our study has some limitations. It is a pilot study that included only 30 patients. However, all of them were healthy individuals meeting relatively strict inclusion/exclusion criteria and the methodology of samples collection was also very meticulous. The pre-analytical phase is extremely important in any oral microbiota study. We decided to observe the medium-term effects of orthodontic appliances; however, it would be interesting to study the dynamics of changes over a long-term period, which was unfortunately not possible in our study. The strongpoints of our study include the complex perspective, examination of clinical indices, oral bacteria, and fungi. At the same time, we employed progressive methods for the qualitative and semiquantitative analysis of oral microbiota.

## Conclusions

To conclude, our study is the first to provide a complex evaluation of the changes in the oral microbiota after the bonding of orthodontic appliances over the medium term. The findings indicate that the placement of appliances can alter the oral environment in this time horizon. We emphasize the importance of the complex perspective in the research of the dynamics of the oral ecosystem as its overall changes could result from the combination of various small, even insignificant differences in oral microbiota. The association between the deterioration of the oral status (represented by the plaque index, which also correlates with the gingival index) in the medium term after the bonding of the orthodontic appliances is much higher for the combined representation of periodontal bacteria and candida species than for the representation of each of these microorganisms separately.

## Supplementary Information

**Additional file 1: Table S1.** Literature review of studies focusing on the effect of fixed orthodontic appliances on oral microbiota, which were not included in the meta-analysis by Guo et al., 2017. **Table S2.** Correlations between the different taste preferences and changes in the oral environment of 30 patients. **Table S3.** The percentages of 3 selected cariogenic and 7 periodontal bacteria relative to the total prokaryotic DNA at two time points – T0 (before bonding of fixed orthodontic appliances) and T1 (till the end of 7^th^ month after bonding of fixed orthodontic appliances) in 30 patients.

## Data Availability

The data of the current study are available from the corresponding author on reasonable request.

## Abbreviations

DMFT: Decay-missing-filled teeth index
PI: Plaque index
GI: Gingival index
GCF: Gingival crevicular fluid
T0: Time point - before bonding of fixed orthodontic appliances
T1: Tme point - during the therapy
N: Number of patients
OR: Odds ratios
WHO: World Health Organisation
MALDI TOF-MS: Matrix-assisted laser desorption ionization-time of flight mass spectrometry
qPCR: Multiplex real-time Polymerase chain reaction
BMI: Body mass index
CFU: Colony-forming units

## References

[1] Proffit WR, Fields HW, Sarver DM (2012). Contemporary orthodontics.

[2] Dewhirst FE, Chen T, Izard J, Paster BJ, Tanner AC, Yu WH, Lakshmanan A, Wade WG (2010). The human oral microbiome. J Bacteriol.

[3] Peterson SN, Snesrud E, Liu J, Ong AC, Kilian M, Schork NJ, Bretz W (2013). The dental plaque microbiome in health and disease. PLoS One.

[4] Sheiham A (2001). Dietary effects on dental diseases. Public Health Nutr.

[5] Shukla C, Maurya RK, Singh V, Tijare M (2016). Evaluation of changes in Streptococcus mutans colonies in microflora of the Indian population with fixed orthodontics appliances. Dent Res J (Isfahan).

[6] Chang H, Walsh LJ, Freer TJ (1999). The effect of orthodontic treatment on salivary flow, pH, buffer capacity, and levels of mutans streptococci and lacto bacilli. Austr Orthod J.

[7] Contaldo M, Lucchese A, Lajolo C, Rupe C, Di Stasio D, Romano A, Petruzzi M, Serpico R. The oral microbiota changes in orthodontic patients and effects on oral health: an overview. J Clin Med. 2021;10(4). 10.3390/jcm10040780. 10.3390/jcm10040780 PMC7919675 33669186

[8] Perkowski K, Baltaza W, Conn DB, Marczyńska-Stolarek M, Chomicz L (2019). Examination of oral biofilm microbiota in patients using fixed orthodontic appliances in order to prevent risk factors for health complications. Ann Agric Environ Med.

[9] Klaus K, Eichenauer J, Sprenger R, Ruf S (2016). Oral microbiota carriage in patients with multibracket appliance in relation to the quality of oral hygiene. Head Face Med.

[10] Maret D, Marchal-Sixou C, Vergnes JN, Hamel O, Georgelin-Gurgel M, Van Der Sluis L, Sixou M (2014). Effect of fixed orthodontic appliances on salivary microbial parameters at 6 months: a controlled observational study. J Appl Oral Sci.

[11] Grzegocka K, Krzyściak P, Hille-Padalis A, Loster JE, Talaga-Ćwiertnia K, Loster BW (2020). Candida prevalence and oral hygiene due to orthodontic therapy with conventional brackets. BMC Oral Health.

[12] Lucchese A, Bondemark L, Marcolina M, Manuelli M (2018). Changes in oral microbiota due to orthodontic appliances: a systematic review. J Oral Microbiol.

[13] Paolantonio M, Pedrazzoli V, di Murro C, di Placido G, Picciani C, Catamo G, De Luca M, Piccolomini R (1997). Clinical significance of Actinobacillus actinomycetemcomitans in young individuals during orthodontic treatment: a 3-year longitudinal study. J Clin Periodontol.

[14] Coronado-Castellote L, Jiménez-Soriano Y (2013). Clinical and microbiological diagnosis of oral candidiasis. J Clin Exp Dent.

[15] Hernández-Solís SE, Rueda-Gordillo F, Flota-Alcocer AD, Agullar-Ayala FJ, Rodríguez-Fernández Mdel S, Lama-González EM (2016). Influence of orthodontic appliances on the occurrence of Candida spp. in the oral cavity. Rev Chilena Infectol.

[16] Tapia CV, Batarce C, Amaro J, Hermosilla G, Rodas PI, Magne F (2019). Microbiological characterisation of the colonisation by Candida sp in patients with orthodontic fixed appliances and evaluation of host responses in saliva. Mycoses..

[17] Sanz-Orrio-Soler I, Arias de Luxán S, Sheth CC (2020). Oral colonization by Candida species in orthodontic patients before, during and after treatment with fixed appliances. A prospective controlled trial. J Clin Exp Dent.

[18] Zheng Y, Li Z, He X (2016). Influence of fixed orthodontic appliances on the change in oral Candida strains among adolescents. J Dent Sci.

[19] Bergamo AZ, de Oliveira KM, Matsumoto MA, Nascimento CD, Romano FL, da Silva RA, da Silva LA, Nelson-Filho P (2019). Orthodontic appliances did not increase risk of dental caries and periodontal disease under preventive protocol. Angle Orthod.

[20] Shukla C, Maurya R, Singh V, Tijare M (2017). Evaluation of role of fixed orthodontics in changing oral ecological flora of opportunistic microbes in children and adolescent. J Indian Soc Pedod Prev Dent.

[21] Löe H (1967). The gingival index, the plaque index and the retention index systems. J Periodontol.

[22] Lochman J, Zapletalova M, Poskerova H, Izakovicova Holla L, Borilova Linhartova P (2020). Rapid multiplex real-time PCR method for the detection and quantification of selected cariogenic and periodontal bacteria. Diagnostics..

[23] Dean A, Arner T, Sunki G, Friedman R, Lantinga M, Sangam S, Zubieta J, Sullivan K, Brendel K, Gao Z (2011). Epi info™, a database and statistics program for public health professionals.

[24] Löe H, Theilade E, Jensen SB (1965). Experimental gingivitis in man. J Periodontol.

[25] Boyd RL (1983). Longitudinal evaluation of a system for self-monitoring plaque control effectiveness in orthodontic patients. J Clin Periodontol.

[26] Guo R, Lin Y, Zheng Y, Li W (2017). The microbial changes in subgingival plaques of orthodontic patients: a systematic review and meta-analysis of clinical trials. BMC Oral Health.

[27] Kouvelis G, Papadimitriou A, Merakou K, Doulis I, Karapsias S, Kloukos D (2021). A prospective cohort study assessing the impact of fixed orthodontic appliances on saliva properties and oral microbial flora. Oral Health Prev Dent.

[28] Arab S, Nouhzadeh Malekshah S, Abouei Mehrizi E, Ebrahimi Khanghah A, Naseh R, Imani MM (2016). Effect of fixed orthodontic treatment on salivary flow, pH and microbial count. J Dent (Tehran).

[29] Rodríguez-Rentería M, Márquez-Preciado R, Ortiz-Magdaleno M, Bermeo-Escalona J, Sánchez-Vargas LO (2021). Frequency of pathogenic microorganisms in removable orthodontic appliances and oral mucosa in children. J Clin Pediatr Dent.

[30] Kim SH, Choi DS, Jang I, Cha BK, Jost-Brinkmann PG, Song JS (2012). Microbiologic changes in subgingival plaque before and during the early period of orthodontic treatment. Angle Orthod.

[31] Naranjo AA, Triviño ML, Jaramillo A, Betancourth M, Botero JE (2006). Changes in the subgingival microbiota and periodontal parameters before and 3 months after bracket placement. Am J Orthod Dentofacial Orthop.

[32] Paolantonio M, Festa F, di Placido G, D’Attilio M, Catamo G, Piccolomini R (1999). Site-specific subgingival colonization by Actinobacillus actinomycetemcomitans in orthodontic patients. Am J Orthod Dentofacial Orthop.

[33] Thornberg MJ, Riolo CS, Bayirli B, Riolo ML, Van Tubergen EA, Kulbersh R (2009). Periodontal pathogen levels in adolescents before, during, and after fixed orthodontic appliance therapy. Am J Orthod Dentofacial Orthop.

[34] Mártha K, Lőrinczi L, Bică C, Gyergyay R, Petcu B, Lazăr L (2016). Assessment of periodontopathogens in subgingival biofilm of banded and bonded molars in early phase of fixed orthodontic treatment. Acta Microbiol Immunol Hung.

[35] Živković-Sandić M, Popović B, Čarkić J, Nikolić N, Glišić B (2014). Changes in subgingival microflora after placement and removal of fixed orthodontic appliances. Srp Arh Celok Lek.

[36] Ristic M, Svabic MV, Sasic M, Zelic O (2008). Effects of fixed orthodontic appliances on subgingival microflora. Int J Dent Hyg.

[37] Choi DS, Cha BK, Jost-Brinkmann PG, Lee SY, Chang BS, Jang I, Song JS (2009). Microbiologic changes in subgingival plaque after removal of fixed orthodontic appliances. Angle Orthod.

[38] Yáñez-Vico RM, Iglesias-Linares A, Ballesta-Mudarra S, Ortiz-Ariza E, Solano-Reina E, Perea EJ (2015). Short-term effect of removal of fixed orthodontic appliances on gingival health and subgingival microbiota: a prospective cohort study. Acta Odontol Scand.

[39] Sallum EJ, Nouer DF, Klein MI, Gonçalves RB, Machion L, Sallum AW, Sallum EA (2004). Clinical and microbiologic changes after removal of orthodontic appliances. Am J Orthod Dentofacial Orthop.

[40] Liu H, Sun J, Dong Y, Lu H, Zhou H, Hansen BF, Song X (2011). Periodontal health and relative quantity of subgingival Porphyromonas gingivalis during orthodontic treatment. Angle Orthod.

[41] Guo L, Feng Y, Guo HG, Liu BW, Zhang Y (2016). Consequences of orthodontic treatment in malocclusion patients: clinical and microbial effects in adults and children. BMC Oral Health.

