# Supplementary material for: Effect of fixed orthodontic appliances on gingival status and oral microbiota: a pilot study
Source: BMC Oral Health. 2022 Oct 27;22:455. doi: 10.1186/s12903-022-02511-9 (PMC9615380; doi:10.1186/s12903-022-02511-9)
Supplement: Supplementary file 1 — Additional file 1: Table S1. Literature review of studies focusing on the effect of fixed orthodontic appliances on oral microbiota, which were not included in the meta-analysis by Guo et al., 2017. Table S2. Correlations between the different taste preferences and changes in the oral environment of 30 patients. Table S3. The percentages of 3 selected cariogenic and 7 periodontal bacteria relative to the total prokaryotic DNA at two time points – T0 (before bonding of fixed orthodontic appliances) and T1 (till the end of 7th month after bonding of fixed orthodontic appliances) in 30 patients. [file 12903_2022_2511_MOESM1_ESM.pdf]

Table S1. Literature review of studies focusing on the effect of fixed orthodontic appliances on oral microbiota, which were not included in the meta-analysis by Guo et al., 2017

**Population**<br>N<br>Mean/median age in years<br>Type of sample<br>PI/GI	**T***<br>(time of the sample collection)	**Oral bacteria**	**Oral candida**	**Reference**
30 patients<br>Age: 12 – 18 years<br>Stimulated saliva samples	T0 = 0<br>T1 = 4 weeks<br>T2 = 12 weeks	All samples were positive for *Neisseria* sp.<br>Following changes were observed, but they were NS.<br>*S. mitis/oralis* increased at T1, but decrease almost to the initial levels at T2<br>*S. anginosus* – a slight decrease at T1<br>*S. mutans* – decrease at T1<br>*S. salivarius* increased a bit at T2<br>*S. sanguinis* decrease at both times, T1 and T2<br>*S. parasanguinis* decrease at T1 and increase at T2<br>Coagulase – negative staphylococci decrease at T1 but increase at T2<br>*Staphylococcus aureus* decrease at T1 and T2	All samples were negative for *Candida albicans*.	(28)
17 patients<br>Age: 11 – 30 years<br>Oral rinses	T0 = 0<br>T1 = 2 weeks<br>T2 = 6 weeks<br>T3 = 12 weeks	NA	Increase of number of the colony forming units of the *Candida* sp. during orthodontic treatment (NS)<br>Dominance of *C. albicans*	(11)
124 patients<br>Mean age 19.5 years	T0 = 0<br>T1 = 1 month<br>T2 = 6 months	NA	NS difference in the presence of *C. albicans* during treatment by an orthodontic appliance.	(17)

Oral swab	T3 = 1 year after treatment<br>T4 = 6 months after completion of ortho. treatment			
50 patients<br>(case/control)<br>Age: 14 – 23 years<br>Oral swabs<br>No statistically significant difference in PI in the group treated with fixed appliances	T = NA	Isolation of various *Streptococci* and *Enterococci* in patients treated with fixed appliances<br>Low prevalence of live protozoans: *Trichomonas tenax* and *Entamoeba gingivalis*<br>Detection of *Acanthamoeba* sp. cyst in several patients treated with fixed appliances	Correlation between the duration of treatment by fixed appliances and frequency of *Candida* sp.<br>Presence of *C. albicans* correlates with poor oral hygiene	(8)
15 patients<br>Age: 11 – 41 years<br>Nonstimulated saliva samples<br>No statistically significant difference in PI and GI	T0 = 0<br>T1 = 30 days<br>T2 = 60 days<br>T3 = 90 days	Purple complex was significantly decreasing from T2<br>Green complex was at the lowest level at T3<br>Yellow and orange complexes decreased significantly only at T3<br>Red complex decreased significantly at T2 and the lowest value was at T3	*Candida* sp. decreased significantly at T2 and the lowest value was at T3	(19)
90 patients<br>Mean age 20.6 ± 7.1 years<br>Oral swabs	T = NA	NA	Fixed orthodontic appliances did not enhance colonization of *Candida* sp.	(16)
75 patients (3 groups, (good oral	T0#^{GOH} = 14.9 ± 7.9 months	*S. mutans* and *Lactobacilli* were detected in all patients	73.4% of the patients were positive for *Candida* sp. in	(9)

hygiene – GOH, poor oral hygiene (POH, POH and white spot lesion – POH/WSL, 25 patients per group)<br><br>Mean age 14.4 ±1.5 years<br><br>Stimulated saliva and plaque samples	T0# POH = 13.4 ± 6.7 months<br><br>T0# POH/WSL = 19.6 ± 11.3 months	Higher prevalence of *S. mutans* in patients with poor oral hygiene (NS)<br><br>Higher prevalence of *Lactobacili*. A significant difference was observed between the GOH and POH/WSL groups	saliva and 60.9 % in dental plaque.<br><br>Significantly lower prevalence of *Candida* sp. in GOH than in POH and in POH/WSL was observed.	
30 patients<br><br>Age: 12 – 18 years<br><br>Saliva sample	T0 = 0<br><br>T1 = 6 weeks<br><br>T2 = 12 weeks<br><br>T3 = 18 weeks	Significant increase of *S. mutans* and *Lactobacillus acidophilus* in T1 and continued up to T2 was observed	Total count of *Candida* sp. *i*ncreased significantly during treatment by orthodontic appliances (starting from T1).<br><br>*C. albicans* was the most predominant.	(29)
50 patients<br><br>Age 10 - 18 years<br><br>Samples via gargle method	T= 1,2,3,6 months before and after the beginning of treatment	NA	Significant increase of *Candida* sp. within 3 months after the installation of the orthodontic appliance	(18)
60 patients<br><br>Age: 13 – 18 years<br><br>Plaque samples	T0 = 0<br><br>T1 = 2 months<br><br>T2 = 3 months	A dramatic increase of *S. mutans* at T1 and T2	Increase of *Candida* sp. at T1 and T2	(5)
60 patients<br><br>(28 patients with fixed orthodontic appliance and 32 with removable appliance)<br><br>Age: 4 – 10 years	T0 = 0<br><br>T1 = 6 months	NA	Significant increase of *Candida* at T1in the group treated with fixed orthodontic appliance<br><br>In the group treated with removable appliances, the increase of *Candida* was not significant<br><br>At T0, the most frequently isolated species was *C. albicans*	(15)

Oral swabs			At T1, the most frequently found species was *C. tropicalis*	
95 patients<br><br>(orthodontic group, 48 patients; control group, without appliances, 47 patients)<br><br>Age: 12 - 16 years<br><br>Stimulated saliva sample	T0 = 0<br><br>T1 = 6 months	Significant increase of *S. mutans* and *Lactobacillus* sp. at T1 in orthodontic group	NA	(10)
60 patients<br><br>(orthodontic group, 30 patients; control group, without appliances, 30 patients)<br><br>Age: 12 – 20 years<br><br>Plaque samples<br><br>Significant difference in PI in orthodontic and control subjects	T1 = 12.4 ± 6.2 months<br><br>T2 = 3 years	Statistically significant greater prevalence of *Aggregatibacter actinomycetemcomitans* in the orthodontic group	NA	(13)

GI, gingival index; GOH, good oral hygiene; NA, not available; NS, non-significant; PI, plaque index; POH, poor oral hygiene; POH/WSL, poor oral hygiene and white spot lesion; ROA, removable orthodontic appliance; *T0, Before bonding of fixed orthodontic appliances; T1, T2, T3, After bonding of fixed orthodontic appliances

The mean wearing time at the time of samples collection; the orthodontic appliance was applied for a significantly longer time in the POH/WSL group than in other groups (p = 0.017)

Table S2. Correlations between the different taste preferences and changes in the oral environment of 30 patients

**Deterioration of**	**PI**	**GI**	**percentage of 7 periodontal bacteria**	**percentage of 3 cariogenic bacteria**	***Candida* sp.**	***Candida* sp. and 7 periodontal bacteria**
**Favorite taste – sweet**						
yes (n=13)	10 (77%)	7 (54%)	8 (62%)	6 (46%)	5 (39%)	10 (77%)
no (n=16)	8 (50%)	4 (25%)	8 (50%)	13 (81%)	0 (0%)	8 (50%)
p-value	0.249	0.143	0.711	0.064	**0.011**	0.249
**Sweetening drinks**						
yes (n=13)	8 (62%)	5 (39%)	6 (46%)	10 (77%)	3 (23%)	8 (62%)
no (n=17)	10 (59%)	6 (35%)	11 (65%)	10 (59%)	2 (12%)	11 (65%)
p-value	1.000	1.000	0.460	0.440	0.628	1.000
**Alcoholic drinks**						
yes (n=9)	5 (56%)	5 (56%)	5 (56%)	5 (56%)	1 (11%)	5 (56%)
no (n=21)	13 (62%)	6 (29%)	12 (57%)	15 (71%)	4 (19%)	14 (67%)
p-value	1.000	0.225	1.000	0.431	1.000	0.687
**Energy drinks**						
yes (n=9)	4 (44%)	1 (11%)	3 (33%)	8 (89%)	1 (11%)	4 (44%)
no (n=21)	14 (67%)	10 (48%)	14 (67%)	12 (57%)	4 (19%)	15 (71%)
p-value	0.418	0.100	0.123	0.204	1.000	0.225

GI, gingival index; PI, plaque index

Table S3. The percentages of 3 selected cariogenic and 7 periodontal bacteria relative to the total prokaryotic DNA at two time points – T0 (before bonding of fixed orthodontic appliances) and T1 (till the end of 7th month after bonding of fixed orthodontic appliances) in 30 patients

**Selected bacteria**	**The difference in % of the total prokaryotic genome in GCF samples between T0 and T1** mean, median (5th, 95th PCTL)	***p*-value**
Streptococcus mutans	0, 0 (-0.02, 0.01)	0.889
Lactobacillus sp.	0, 0 (-0.02, 0.01)	0.136
Actinomyces sp.	0.85, 0.44 (-1.10, 5.52)	**0.027**
**All 3 cariogenic bacteria**	0.84, 0.44 (-1.10, 5.45)	**0.030**
Aggregatibacter actinomycetemcomitans	-0.02, 0 (-0.04, 0.01)	0.866
Porphyromonas gingivalis	0, 0 (0, 0)	1.000
Tannarella forsythia	0, 0 (0, 0)	1.000
Treponema denticola	0, 0 (0, 0)	0.317
Parvimonas micra	0, 0 (0, 0)	0.317
Prevotella intermedia	0, 0 (0, 0)	0.314
Fusobacterium nucleatum	0.95, 0.13 (-2.98, 8.98)	0.339
**All 7 periodontal bacteria**	0.94, 0.13 (-2.98, 8.98)	0.329
**All 10 bacteria**	1.78, 0.47 (-2.04, 11.75)	0.086

T0, before bonding of fixed orthodontic appliances; GCF, gingival crevicular fluid; please note that when sampling GCF, dental plaque is sampled as well

p-value: The difference between T1 and T0 measurements is evaluated using the Wilcoxon matched-pairs signed-rank test.
